# Prognostic utility of mid-regional pro-adrenomedullin and growth differentiation factor 15 in patients undergoing transfemoral transcatheter aortic valve implantation

**DOI:** 10.1007/s00392-024-02560-w

**Published:** 2024-10-25

**Authors:** Kerstin Piayda, Stanislav Keranov, Luisa Schulz, Mani Arsalan, Christoph Liebetrau, Won-Keun Kim, Felsix J. Hofmann, Pascal Bauer, Sandra Voss, Christian Troidl, Samuel T. Sossalla, Christian W. Hamm, Holger M. Nef, Oliver Dörr

**Affiliations:** 1 Department of Cardiology, Justus-Liebig-University Giessen, Medical Clinic I, Giessen, Germany; 2 Medical Faculty, Department of Cardiothoracic Surgery, Goethe-University Frankfurt, Frankfurt, Germany; 3 Cardioangiologisches Centrum Bethanien (CCB), Im Prüfling 23 60389, Frankfurt Am Main, Germany; 4 Department of Cardiology, Kerckhoff-Klinik, Bad Nauheim, Germany; 5 Kerckhoff Herzforschungsinstitut, Bad Nauheim, Germany; 6 German Center for Cardiovascular Research (DZHK), Partner Site Rhine-Main, Bad Nauheim, Germany; 7 Heart and Vascular Center Bad Segeberg, Cardiology and Angiology, Segeberger Kliniken GmbH, Bad Segeberg, Germany

**Keywords:** TAVI, Survival, Biomarkers, Mid-regional pro-adrenomedullin, Growth differentiation factor 15

## Abstract

**Background:**

Risk prediction in patients with severe, symptomatic aortic stenosis (AS) undergoing transcatheter aortic valve implantation (TAVI) remains an unsolved issue. In addition to classical risk scoring systems, novel circulating biomarkers like mid-regional pro-adrenomedullin (MR-proADM) and growth differentiation factor 15 (GDF-15) may be of value in assessing risk.

**Methods:**

Consecutive patients undergoing elective transfemoral TAVI were included in this prospective observational study. Baseline information, imaging findings, blood samples, and clinical outcomes were collected. Blood levels of the classical biomarkers interleukin-6 (IL-6) and high-sensitivity C-reactive peptide (hsCRP) and of the novel biomarkers MR-proADM and GDF-15 were measured and their predictive utility for mortality assessed.

**Results:**

The study cohort consisted of 92 patients undergoing TAVI. The median age was 80.7 years [IQR 77.2;83.3], and 48 (52.2%) were male. Analysis of the area under the curve (AUC) of the receiver-operating characteristics showed that the hsCRP levels discriminated poorly (AUC 0.66, 95% CI [0.52;0.8], *p* = 0.027), whereas all other biomarkers reached a higher level of discrimination (IL-6: AUC 0.76, 95% CI [0.66;0.86], *p* < 0.001; MR-proADM: AUC 0.73, 95% CI [0.61;0.85], *p* = 0.002; GDF-15: AUC 0.73, 95% CI [0.61;0.85], *p* = 0.002). Kaplan–Meier analysis in conjunction with Youden J-statistics yielded the optimal cutoff points for each biomarker to predict survival: IL-6 4.65 pg/mL, hsCRP 12.9 mg/L, MR-proADM 1.02 nmol/L, and GDF-15 2400.1 pg/mL.

**Conclusion:**

Novel circulating biomarkers like MR-proADM and GDF-15 may provide additional value in predicting survival after TAVI.

## Introduction

Aortic valve stenosis (AS) is a common valvular disorder in the general population [1] and is predominantly present in the elderly [2]. The estimated prevalence of AS in patients ≥ 75 years old is 4.9 million in Europe and 2.7 million in North America [3]. In patients with symptomatic, severe AS, transcatheter aortic valve implantation (TAVI) presents a valuable therapeutic option whose indication nowadays has been widened to an intermediate- and low-risk patient clientele [4–6]. Computational modeling predicted that approximately 189 836 patients could benefit from TAVI in Europe, and the number of potential candidates increases by 9 189 annually (95% CI: 3 898;16 681) [3].

Although TAVI is an established treatment option, the procedure does not come without risks. Vascular complications, stroke, renal failure, or higher-grade conduction disturbances remain problematic, [7] and post-TAVI risk modeling remains an unresolved issue [8, 9]. Novel circulating biomarkers may provide information about distinct pathophysiological phenotypes of disease, potentially paving the way for personalized medicine, and they may enhance the accuracy of mortality prediction. In this context, mid-regional pro-adrenomedullin (MR-proADM) and growth differentiation factor 15 (GDF-15) have thus far only been investigated to a small extent. MR-proADM has mostly been studied in the setting of chronic heart failure [10, 11]. The adrenomedullin system can serve as a marker to quantify systemic microvascular dysfunction: in volume overload, adrenomedullin secretion is increased as a compensatory mechanism to hinder tissue congestion and to ensure cell barrier function [12]. MR-proADM is a stable precursor of the bioactive adrenomedullin and does not differentiate between the biologically active and non-active forms [13]. GDF-15 has mostly been studied in the context of heart failure and has been shown to provide good prognostic information [14, 15]. It belongs to the transforming growth factor-ß cytokine superfamily and its expression by myocytes increases in settings of oxidative stress, ischemia, mechanical stretch, or angiotensin II stimulation [16]. These pathophysiological pathways may be of interest in patients with severe AS, but have not yet been investigated in detail.

Hence, we examined the potential prognostic role of MR-proADM and GDF-15 in relation to other biomarkers in a patient cohort with severe AS undergoing TAVI.

## Methods

### Study population

Patients with severe, symptomatic AS who were indicated for TAVI by the local heart team were enrolled in this prospective, observational study from July 2017 to September 2019 at the Giessen Heart Center. Baseline information, imaging findings, blood samples, and clinical outcomes were collected. Follow-up was conducted via telephone interview at 6 months and 1, 2, and 3 years post-TAVI to assess functional status and survival. An in-person follow-up assessment including blood sample collection and a transthoracic echocardiogram was performed 6 months after the procedure. Exclusion criteria were systemic infection, active malignancy, or immunosuppressive therapy.

### Biomarker analysis

Venous blood samples (MR-proADM and GDF-15) were collected in plain tubes, and all measurements were carried out batch-wise on thawed serum samples by enzyme-linked immunosorbent assay conducted by experienced staff blinded to patient characteristics. The mean minimum detectable level for the GDF-15 assay is 2.0 pg/ml (GDF-15 assay, Quantikine ELISA, R&D Systems, Abingdon, UK. Standard assays were carried out to measure the more established biomarkers, high-sensitive C-reactive protein (hsCRP), interleukin-6 (IL-6), and brain natriuretic peptide. Twenty healthy patients served as the control group.

### Statistical analysis

Categorical data are displayed as numbers (n) and percentages. Continuous variables are presented as mean with standard deviation and non-parametric variables are shown as median with interquartile ranges (IQR, 25th to 75th quartiles). The Kolmogorov–Smirnov test was used to test for normal distribution. Independent groups were compared with Student’s *t* test, Mann–Whitney *U* test, Wilcoxon test, or Chi-squared test, as appropriate. The prognostic value of biomarkers was assessed by analyzing receiver-operating characteristics (ROC) curves. The combined predictive value of classical risk predictors and the here investigated biomarkers for mortality after TAVI were calculated by binary logistic regression. ROC comparison between pre-defined panels was assessed with the DeLong method. Survival was analyzed by Kaplan–Meier estimation. The best cutoff points to predict survival for each biomarker were defined by Kaplan–Meier analysis in conjunction with Youden J-statistics. Data were analyzed and graphically displayed with SPSS Version 29 (IBM, Armonk, NY, USA).

### Ethics

Ethical oversight was secured. This clinical investigation was in line with the Declaration of Helsinki. The local institutional review board approved the study (AZ: 99/13). All patients provided written informed consent prior to inclusion.

## Results

### Study population and baseline findings

A total of 92 consecutive patients were included between July 2017 and September 2019. The median age was 80.7 years [77.2;83.3] and 48 (52.2%) were male. Patients had an elevated cardiovascular risk profile: diabetes mellitus was present in 28 (30.4%), hypercholesterinemia in 40 (43.5%), and hypertension in 75 (80.4%) patients. Over half of the patients (n = 60, 65.1%) presented with New York Heart Association class III or IV symptoms, and patients were judged to be at an intermediate surgical risk (EuroSCORE II: 6.4% [4.4; 10.7]). Left ventricular function was preserved in 55% [46;60], and the median aortic valve area was 0.77 cm^2^ [0.6;0.9]. Further baseline characteristics are presented in Table 1.

**Table 1:** Baseline characteristics of the entire cohort (n = 92)

Parameter	Value^a^
Age, years	80.7 [77.2;83.3]
Sex, male	48 (52.2)
Coronary artery disease	68 (73.9)
EuroSCORE II	6.4 [4.4;10.7]
NYHA class III and IV	60 (65.2)
Cardiovascular risk factors	
- Diabetes mellitus	28 (30.4)
- Hypertension	75 (80.4)
- Hypercholesterinemia	40 (43.5)
Laboratory findings	
- eGFR, mL/min/1.73 m^2^	67.5 [50.4;85.5]
Echocardiographic findings	
- Aortic valve area, cm^2^	0.77 [0.6;0.9]
- Mean pressure gradient, mmHg	37.5 [29.3;47]

^a^Values represent n (%) or median [IQR]

Abbreviations: eGFR: estimated glomerular filtration rate, NYHA: New York Heart Association

Patients in the control group were younger (74.6 years [69.6;78.4]) and predominantly male (n = 14 (70%), *p* = 0.092).

### TAVI and follow-up

TAVI was performed successfully in all patients. Acute kidney injury was observed in 16 (17.4%) patients. Vascular and bleeding complications occurred in 10 (10.8%) and 28 (30.4%) patients, respectively. One major stroke was identified in the study population, and permanent pacemaker implantation was necessary in 14 (15.2%) patients.

Of note, if patients were stratified by survival during follow-up, only bleeding complications were significantly more frequent in deceased patients as compared to those who survived (alive: 17/68 (25%) deceased: 11/24 (45.8%), *p* = 0.006).

### Mortality prediction and biomarker analysis

**Fig. 1:**
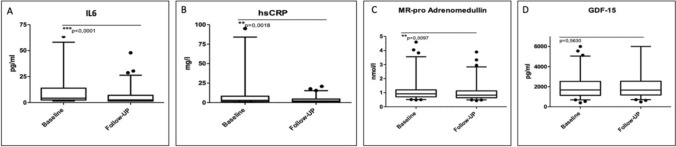
Biomarker levels of IL-6, hsCRP, MR-proADM, and GDF-15 in patients at baseline and during follow-up

The median clinical follow-up period was 620 days. Within this time period, 24 (26.1%) patients died. Patients who died during follow-up had significantly higher creatinine levels (alive: 0.95 mg/dL [0.8;1.2] vs. deceased: 1.1 mg/dL [0.9;1.5], *p* = 0.027) and brain natriuretic peptide (BNP) levels (alive: 218.5 pg/mL [127.3;875.8] vs. deceased: 646 pg/mL 154;1349]) at baseline as compared to the survivors. Patients were stratified by survival in the follow-up period, and baseline biomarker levels were compared between survivors and deceased patients, and a control group (Table 2). All biomarker levels were significantly elevated in deceased patients as compared to survivors. Compared to the control group, the whole study population had higher baseline levels of circulating IL-6, hsCRP, MR-proADM, and GDF-15 (Table 2). During follow-up, IL-6, hsCRP, and MR-proADM decreased significantly, whereas GDF-15 levels remained relatively unchanged (Fig. 1).

**Table 2:** Biomarker levels of the study population stratified by survival and of the control group

Biomarker	Survivors^a^	Deceased	p value	Control group	p value (alive vs. control)	p value (deceased vs. control)
IL-6, pg/mL	4.2 [2.5;13.8]	14.4 [7.5;42.1]	< 0.001	1.8 [1.5;3.4]	< 0.001	< 0.001
hsCRP, mg/L	2.9 [1.2;8.2]	5.3 [2.2;26.7]	0.022	1.5 [1;2.5]	0.013	< 0.001
MR-proADM, nmol/L	0.92 [0.7;1.2]	1.3 [1;1.6]	< 0.001	0.66 [0.52–0.86]	0.004	< 0.001
GDF-15, pg/mL	1675.2 [1141;2524]	2770 [2401;3701]	0.001	967.6 [723;1286]	< 0.001	< 0.001

^a^Values represent median [IQR]

Abbreviations: hsCRP, high-sensitivity C-reactive protein; GDF-15, growth differentiation factor 15; IL-6, interleukin-6; MR-proADM, mid-regional pro-adrenomedullin

**Fig. 2:**
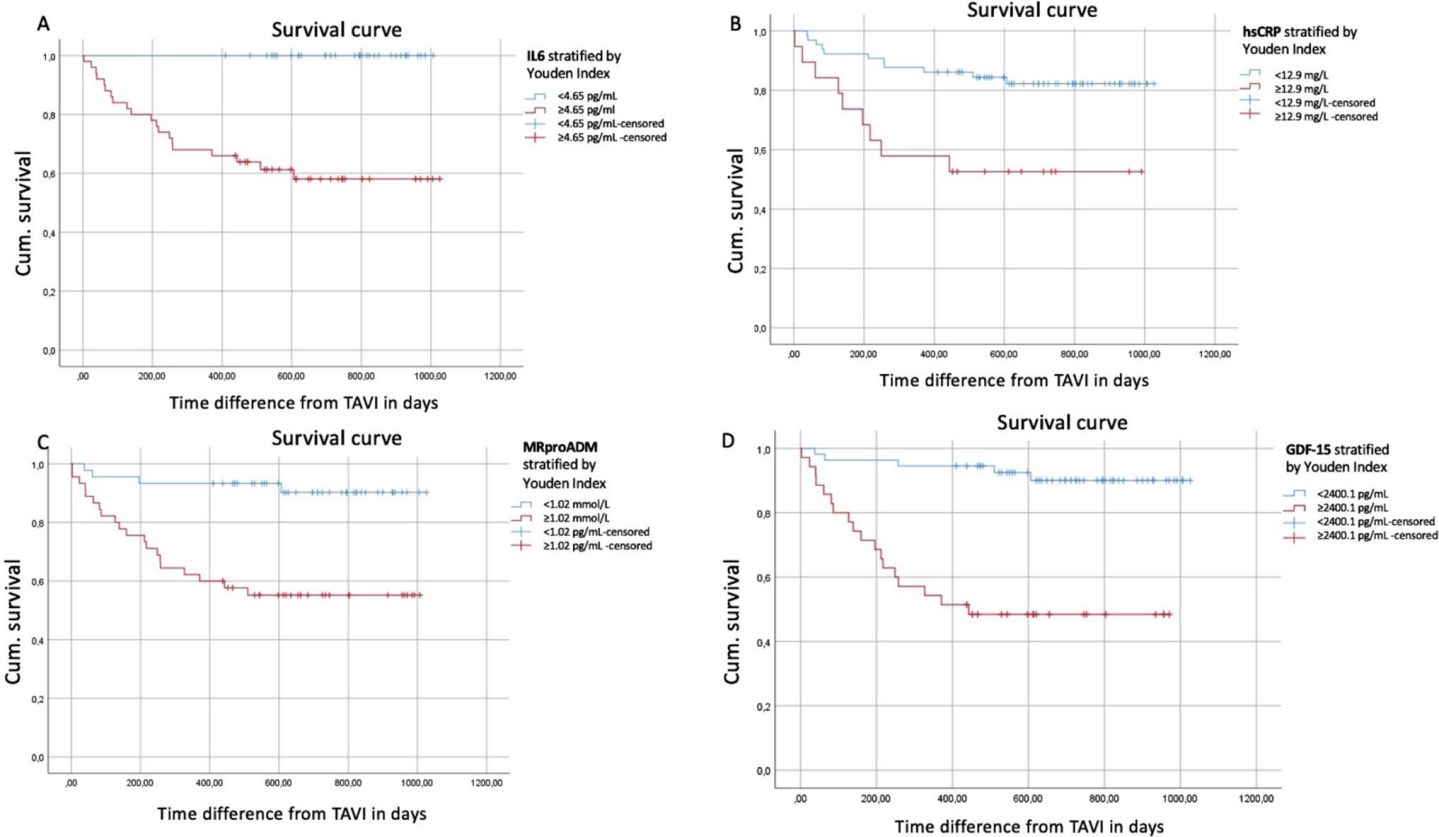
Survival analysis with Youden Index to predict the best cutoff value for each biomarker

**Fig. 3:**
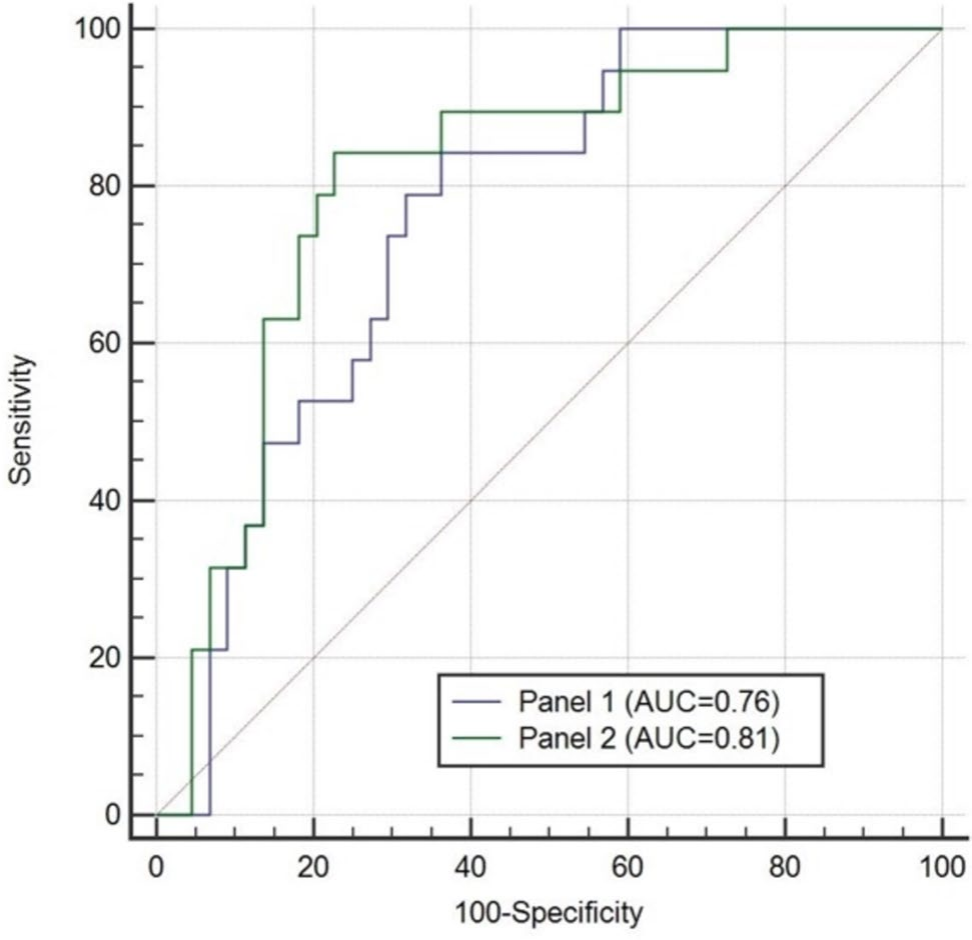
Receiver-operating caracteristics comparsion between Panel 1 BNP + eGFR + Euroscore II) and Panel 2 (BNP + eGFR + Euroscore II + GDF-15 + MRproADM)

In a receiver-operating characteristics (ROC) analysis, hsCRP levels showed a poor discrimination (AUC 0.66, 95% CI [0.52;0.8], *p* = 0.027) with survival, whereas other biomarkers reached a higher level of discrimination (IL-6: AUC 0.76, 95% CI [0.66;0.86], *p* < 0.001; MR-proADM: AUC 0.73, 95% CI [0.61;0.85], *p* = 0.002; GDF-15: AUC 0.73, 95% CI [0.61;0.85], *p* = 0.002). Additionally, ROC analysis was performed for established risk predictors such as BNP (AUC 0.643), eGFR (AUC 0.637) and EuroScore II (AUC 0.523), which all showed poor discrimination for mortality after TAVI. In a next step, binary logistic regression with BNP, eGFR, EuroSCORE II, GDF-15, and MRproADM was performed (Table 3). The combined predictive value of classicial risk stratification markers (Panel 1: BNP + eGFR + Euroscore II), and classical risk markers in conjunction with the here investigated new biomarkers (Panel 2: BNP + eGFR + Euroscore II + GDF-15 + MRproADM) was calculated Fig. 2. The combined predictive value of Panel 1 was 0.762 (standard error 0.06, 95% CI [0.638;0.860]), which corresponds to an acceptale level of discrimination. If novel biomarkers were added (Panel 2), the AUC increased to 0.811 (standard error 0.05; 95% CI [0.693;0.899)], which corresponds to an excellent correlation. In an ROC comparison analysis, no statistical significane between Panel 1 and 2 could be determined (*p* = 0.417, Fig. 3).

**Table 3:** Binary logistic regression for mortality prediction after TAVI

Variable	Regression coeffiecent B	Standard error	Wald	Significance	Exponent (B)	95% confidence interval for Exp (B)
BNP, pg/mL	0.001	< 0.001	2.59	0.107	1.001	[1.00;1.001]
eGFR, mL/min/m^2^	0.002	0.015	0.026	0.873	1.002	[0.974;1.032]
EuroScore II, %	-0.190	0.084	5.12	0.023	0.827	[0.701;0.975]
MR-proADM, nmol/L	0.002	0.002	1.02	0.311	1.002	[0.998;1.006]
GDF-15, pg/mL	0.001	< 0.001	4.83	0.028	1.001	[1.000;1.001]

Abbreviations: BNP, brain natriuretic peptide; eGFR, estimated glomerular filtration rate, EuroScore II: European System for Cardiac Operative Risk Evaluation; GDF-15, growth differentiation factor 15; MR-proADM, mid-regional pro-adrenomedullin

Kaplan–Meier analysis in conjunction with the Youden J-statistics yielded the optimal cutoff points for each biomarker to predict survival: IL-6 4.65 pg/mL, hsCRP 12.9 mg/L, MR-proADM 1.02 nmol/L, and GDF-15 2 400.1 pg/mL. Corresponding Kaplan–Meier curves are displayed in Fig. 2.

## Discussion

The current analysis of 92 consecutive patients undergoing transfemoral TAVI showed that the novel circulating biomarkers MR-proADM and GDF-15 (1) have predictive value for survival comparable to that of IL-6 and (2) may be of additional value to optimize risk prediction in conjunction with already established risk predictors such as BNP, eGFR, and EuroSCORE II in the future.

TAVI has become a cornerstone therapy in the management of patients with severe AS. In daily clinical practice, several risk scoring systems are used to predict mortality and support informed decision-making. European and American guidelines on the management of valvular heart disease [17, 18] use conventional surgical risk scores, like the European system for cardiac operative risk evaluation II (EuroSCORE II [19]) or the Society of Thoracic Surgeons predicted risk of mortality (STS PROM [20]) score, to facilitate treatment recommendations. However, these scores overestimate mortality after TAVI by a wide margin [21, 22]. Even specifically developed risk scoring systems for TAVI, like the STS/ACC TAVI score [23], the TAVI2-SCORE [24], or the GARY risk score [25], have failed to provide an accurate estimation of mortality. In this context, biomarkers may be of additional informative value. Classical biomarkers, such as N-terminal proBNP or BNP [26, 27] and troponin [28, 29], have been widely studied to monitor congestion and myocardial injury in patients undergoing TAVI. However, these biomarkers do not specifically account for the underlying pathophysiological pathways and are not specific for the disease entity. Disease-specific biomarkers, in addition to genotyping [30], may enhance precise and individualized patient care within the emerging concept of personalized medicine.

ADM is a secretory product of the vascular endothelium and showed better discriminative value than N-terminal proBNP or BNP in patients with acute and chronic heart failure [31, 32]. Its precursor MR-proADM has been investigated to a limited extent in patients with AS: blood levels are elevated in patients with AS and are approximately in the same range as in patients with acute heart failure [33, 34]. Tan et al. studied its predictive value in individuals with moderate to severe AS and showed that among other biomarkers like IL-6, hsCRP, N-terminal proBNP, and high-sensitivity troponin, MR-proADM had the highest discriminative value to predict all-cause mortality, hospitalization for heart failure, and progression to NYHA class III/IV symptoms [35]. In other investigative settings, MR-proADM was used in a combined biomarker analysis to predict adverse events after TAVI [36]. It has also been used in addition to common surgical risk scores such as the EuroSCORE II to improve survival prediction [37].

GDF-15 has mostly been investigated in the setting of coronary artery disease and heart failure [38–40]. To our knowledge, only two other studies examined its role in patients with AS. Basmadjian et al. [41] showed that elevated GDF-15 levels were associated with frailty, ventricular dysfunction, and lower functional capacity in patients with moderate to severe AS and preserved ejection fraction. Interestingly, in a small-scale study by Fabiani et al., GDF-15 also correlated with frailty and echocardiographic parameters (indexed left atrial volume and pulmonary artery pressure) in patients undergoing TAVI [42]. Hence, this biomarker might provide additional clinical information that is not captured in classical risk scores.

Our findings are in line with the current body of evidence. In addition to classical risk sores and biomarkers that have already entered widespread clinical use, MR-proADM and GDF-15 can be of additional value to predict survival after TAVI. This approach may further be refined by assessing advanced imaging parameters and modalities to take into consideration staged cardiac damage classification and myocardial stress and workload. Additionally, MR-proADM and GDF-15 may have a role in predicting the optimal time frame for intervention in asymptomatic, severe aortic stenosis since those biomarkers are more disease specific than classical biomarkers suchs BNP. However, this statement is speculative and warrants further investigation.

### Limitations

This is only a single-center experience with a small study population. Larger-scale studies with extended follow-up are needed to elucidate the role of MR-proADM and GDF-15 as prognostic biomarkers for patients undergoing TAVI. The relatively small size of the derivation cohort data necessitates validation in an external cohort.

## Conclusion

Novel circulating biomarkers such as MR-proADM and GDF-15 may be of additional value to predict survival after TAVI.

## Abbreviations

AS: Aortic stenosis
AUC: Area under the curve
EuroSCORE II: European System for Cardiac Operative Risk Evaluation
hsCRP: High-sensitivity C-reactive protein
GDF-15: Growth differentiation factor 15
IL-6: Interleukin-6
NYHA: New York Heart Association
MR-proADM: Mid-regional pro-adrenomedullin
ROC: Receiver-operating characteristics
STS PROM: Society of Thoracic Surgeons predicted risk of mortality score
TAVI: Transcatheter aortic valve implantation
